# Diversity of fungal flora in raw milk from the Italian Alps in relation to pasture altitude

**DOI:** 10.1186/2193-1801-2-405

**Published:** 2013-08-27

**Authors:** Simona Panelli, Eva Brambati, Cesare Bonacina, Maria Feligini

**Affiliations:** Istituto Sperimentale Italiano Lazzaro Spallanzani, Località La Quercia, Rivolta d’Adda, (Cremona), 26027 Italy

**Keywords:** Raw milk, Yeasts, Moulds, Alpine pastures, Barcodes

## Abstract

The present paper explores the diversity of mycobiota inhabiting raw milk sampled at different altitudes (1400 m, 1800 m, 2200 m) from cows grazing Alpine pastures of Valle d’Aosta (North-Western Italian Alps). To this aim, multilocus sequencing was performed at barcodes commonly used for fungal identification (ITS1, D1/D2 domains of the 26S rRNA gene, and part of the β-tubulin gene). A total of 31 species were detected, most of them yeasts, followed by moulds and by 2 sequences of macroscopic fungi. Several yeasts and moulds were well-characterized inhabitants of the dairy environment, known to positively contribute to cheesemaking. Among these, *Candida* was the most represented genus with a tendency to cluster at the highest altitudes (6 over 8 observations at ≥ 1800 m), and *Kluyveromyces marxianus* the most abundant single species, retrieved at all altitudes. The environmental ascomycetous *Atrotorquata lineata,* never put in relation with food nor described outside North-America, was another species among those most frequently retrieved and was detected in 6 milks at 1400 and 1800 m. The remaining fungi, in general never reported in milk, were mostly environmental. Many of them resulted associated with plants as pathogens or symbionts. Finally, the highest sampled altitude yielded a significant fungal diversity (17 species). This work enlarges the knowledge of fungal consortia inhabiting raw milk and introduces microbial ecology among the altitude-dependent factors, in the composition of Alpine pastures, with the potential of shaping the properties of milks and cheeses, together with the already described physical, chemical and botanical variables.

## Introduction

In all cheeses made from raw milk, the contribution to flavouring of factors linked to the area of production is prominent. The specific environmental conditions (e.g., those of valleys and Alpine pastures) often linked to the use of traditional cheesemaking processes, define cheese aroma and texture by determining the unique features of the raw milk (Martin et al. 2005). These comprehend (i) chemical characteristics and presence of specific molecules (e.g., carotenes, terpenes) that significantly depend on grass botanical composition and on geographical variants as altitude (Buchin et al. 1999); (ii) composition in natural micro- and mycobiota. The contribution to cheese homogeneity of autochthonous lactic micro- and mycoflora is deep (Beresford et al. 2001): for this reason, production regulations currently allow the exclusive addition to raw milk of bacterial strains isolated in the area of production. Therefore, the description of the microbial communities inhabiting raw milk and cheeses which production is allowed only within well-defined geographical areas is particularly relevant for dealing with product quality features and authenticity assurance. These are relevant points because the production of raw milk cheeses is growing, and consumers more and more seek organic foods and traditional sensory characteristics (Laurenčic et al. 2008).

Bacterial species inhabiting raw milk have been recently reported by our group (Giannino et al. 2009) in a context where the microbial diversity characterizing traditional raw milk cheeses and the milks used for their production is still largely uncharacterized. This is especially true for eukaryotic microorganisms, whose importance is being increasingly acknowledged in spite of a continuously evolving classification. They in fact play a synergistic role by excreting growth factors as vitamins, and by priming the growth of acid-sensitive bacteria thanks to their ability of metabolizing lactate, which causes a shift of the pH towards neutrality (Butinar et al. 2005). Fungal flora is also able of generating components of aroma (e.g., amino acids, fatty acids, esters) that significantly contribute to flavour (Lund et al. 1995; Vasdinyei & Deak 2003). On the other hand, mycobiota can act as spoilage organisms that contribute to the deterioration of sensorial properties (Fleet 2007), or pose relevant medical problems, as in the case of micotoxins produced by moulds (Filtenborg et al. 1996).

In the present paper, the mycobiota of cow raw milk from highaltitude pastures used for Fontina cheese production are characterized using DNA barcoding. Samples come from several Alpine valleys within Valle d’Aosta region (Italian Alps) and are representative of different altitudes (altitude equal to or higher than 1400 m above sea level).

Fontina is a PDO cheese exclusively produced in Valle d’Aosta region under the Commission Regulation 1107 ⁄ 96 ⁄ EC and later amendments. It is a semi-cooked, smear-rind cheese produced using full cream raw milk derived from a single milking of cows belonging exclusively to the autochthonous Valdostana breed.

DNA barcoding is the culture-independent approach considered as the election technique for fungal species identification (Begerow et al. 2010; Giraud et al. 2010). The barcodes considered in the present paper are both ribosomal (the internal transcribed spacer 1, ITS1, between the 18S rRNA and 5.8 rRNA genes, and the variable D1/D2 domains at the 5’ end of the 26S rRNA gene) and non-ribosomal (β-tubulin, TUB). Barcoding was performed on DNA directly extracted from milk without intermediate culturing and generation of fungal isolates to avoid disadvantages inherently linked to this step.

## Methods

### Sampling

A total of 40 bulk raw milks were collected during summer (June-July-August) from 1400 m, 1800 m, 2200 m Alpine pastures across Valle d’Aosta region (North-Western Italian Alps). In addition, 9 raw milks from different herds were sampled at an altitude <700 m (bottom of valleys). After sampling, milk was frozen at −20°C.

### DNA extraction

DNA was isolated from all samples by phenol-chloroform extraction. Raw milks (40 ml-each) were thawed at 37°C for 2 h and centrifuged at 1,000 ×*g* for 10 min at 4°C. Pellets were picked up with 1 ml lysis buffer (10 mM Tris–HCl pH 7.5, 1 mM EDTA, 51 mM NaCl, 2 mg/ml SDS) and digested with 50 mg proteinase K (Fermentas Life Sciences, Burlington, ON, Canada) on a linear shaker overnight at 42°C. After the addition of 50 mg lysozyme solution (Sigma-Aldrich, St. Louis, Mo., USA), samples were incubated for 1 h at 37°C. 1 ml of phenol was added to the digested suspensions, that were then mixed for 10 min and centrifuged at 11,000 ×*g* for 10 min at 4°C. Supernatants were transferred in sterile tubes and solvent extraction was repeated 3 more times using 1 : 1 phenol–chloroform mix. After addition of 0.1 volumes sodium acetate (pH 5.2) and 2.5 volumes ethanol, samples were kept at – 20°C overnight. DNA was pelleted by centrifugation (11,000 ×*g* for 20 min at 4°C) and suspended in 50 μl of double distilled sterile water. Its concentration and purity were assessed using a NanoDrop® ND-1000 spectrophotometer (NanoDrop Technologies, Inc., Wilmington, DE, USA).

### Barcodes amplification

All PCR reactions were set up in a 50 μl volume containing 10 mM Tris–HCl pH 8.3, 50 mM KCl, 0.2 mM dNTPs, 2.5 mM MgCl_2_, 1 μM of each primer, 3.5 U AmpliTaq Gold (Life Technologies, Foster City, CA), and about 50 ng template genomic DNA.

Primers used in this study are the following: (i) for the ITS1 barcode: forward primer ITS1 (TCCGTAGGTGAACCTGCGG), reverse primer ITS2 (GCTGCGTTCTTCATCGATGC) that give PCR products of variable sizes in different species (White et al. 1990); (ii) for the D1/D2 barcode: forward primer NL1 (GCATATCAATAAGCGGAGGAAA), reverse primer NL4 (GGTCCGTGTTTCAAGACGG) that give PCR products around 600 bp, with slight species-specific variations (Kurtzmann & Robnett 1997); (iii) for the TUB barcode: forward primer BT2a (GGTAACCAAATCGGTGCTGCTTTC), reverse primer BT2b (ACCCTCAGTGTAGTGACCCTTGGC) that give amplicons around 500 bp (Glass & Donaldson 1995).

Amplifications were performed on a Bioer LifePro thermal cycler (Bioer Technology Co, Ltd, Tokyo, Japan) according to the following cycling program: initial denaturation step (95°C 10 min), 35 cycles at 94°C 30 s, 56°C 30 s, 72°C 40 s, final elongation (72°C 10 min). Only for D1/D2 the annealing was 60°C 1 min, the extension 72°C 1 min and the number of cycles 30. Samples were analyzed using all three barcodes. DNA from milk sampled at bottom of valleys were amplified at the D1/D2 barcode.

### Cloning and sequencing

PCR products were visualized on 2% TBE agarose gels. Amplicons were either sequenced directly on both strands using an ABI PRISM 3130 ×l automated DNA sequencer (Life Technologies) or, in the case of faint or multiple bands, subjected to an intermediate cloning step using the TOPO ® TA cloning kit (Life Technologies). In detail: 3 μl of PCR products were ligated into the pCR®4-TOPO® (Life Technologies). Recombinant plasmids were used to transform *Escherichia coli* Top10 competent cells according to the manufacturer’s protocol. After plating onto kanamycin-LB plates, transformant clones were picked and subjected to colony PCR using M13 primers (F: TTTCCCAGTCACGACGTTGTA; R: TTGTCAGCGGATAACAATTTC). For each cloning reaction, amplicons representing recombinant inserts of the expected molecular weight were purified using Agencourt AMPure XP-PCR purification beads (Beckam Coulter, Brea, CA) and sequenced on an ABI PRISM 3130 ×l automated DNA sequencer (Life Technologies) using T7 primer (TAATACGACTCACTATAGGG). Three recombinant plasmids were normally sequenced per cloning reaction. In the case of multiple bands, this number was raised up to 10.

### Sequence analysis

Electropherograms were visually inspected and manually edited for quality using the Bioedit program (http://www.mbio.ncsu.edu/BioEdit/BioEdit.html) (Hall 1999). Sequences were then compared to the GenBank database by means of BLAST searches (http://www.ncbi.nlm.nih.gov/BLAST). When necessary, sequences were aligned using the ClustalW algorithm implemented in the Mega5 software (Kumar et al. 2004). RNA secondary structures of domains D1/D2 encoded by 26S rRNA genes with different in-del polymorphisms were predicted using the following web servers: (i) mfold web v 2.3 (http://mfold.rna.albany.edu), with the default parameters, except for the temperature, that was set at 25°C; (ii) RNAalifold (http://rna.tbi.univie.ac.at/cgi-bin/RNAalifold.cgi) that predicts the consensus secondary structure of aligned sequences.

## Results

### Fungal diversity in relation to altitude

Table 1 reports the list of the fungal species detected at the various altitudes by multilocus gene sequencing. All the sequences not resolved at least at the genus level have been omitted as well as the cases that yielded as only hit an homology with “unidentified” or “uncultured” fungi, without any taxonomic details. Other non included results comprehend a conspicuous number of bacterial, plant and metazoan species, consistently identified by all the barcodes, either in the case of direct sequencing and when sequencing cloned amplicons. Some of these sequences are mentioned in the Discussion.

**Table 1:** **Fungal species identified in raw bulk milk sampled at various altitudes**

Altitude	Milks from individual pastures (n***)***	Identity	Source and significance	References
1400	5	*Kluyveromyces marxianus*	Raw milk, yogurt, cheese. Potential probiotic	(Hatoum et al. 2012; Jacques & Casaregola 2008; Lane & Morrisey 2009)
		*Debaryomyces hansenii*	Marine environment, brine, cheese, floor, walls, hands and equipments in cheese plants. Potential probiotic	(Hatoum et al. 2012; Jacques & Casaregola 2008; Welthagen & Viljoen 1998)
		*Galactomyces geotrichum*	Soil, insects, milk, cheese. Plant pathogen	cbs.knawl.nl
		*Atrotorquata lineata*	Associated to salt-tolerant plants	(Kohlmeyer & Volkmann-Kohlmeyer 1993)
		*Aspergillus penicilloides*	House dust, food, water. Produces potential antibiotics	(Itabashi et al. 2006)
		*Colletothricum gloesporioides*	Soil. Pathogen for crops	(Cai et al. 2009)
		*Pichia membranifaciens*	Cheese, wine, brines, spoiled food, sugar cane, tequila, plants	(Corbo et al. 2001; Jacques & Casaregola 2008)
		*Exophiala pisciphila*	Soil, water, plants, decaying wood material	(Figel et al. 2013)
		*Ophiocordyceps oxycephala*	Entomopathogen for wasps	cordyceps.us/node/5820
		*Alternaria solani*	Soil, roots. Pathogen for tomatoes and potatoes	(Ai et al. 2012)
		*Phoma* spp*.*	Air, spoiled vacuum-packaged cheese	(Hocking & Faedo 1992)
		Unknown fungus	Soil	Acc.No. JQ311712
1800	11	*Kluyveromyces marxianus*	Raw milk, yogurt, cheese. Potential probiotic	(Hatoum et al. 2012; Jacques & Casaregola 2008; Lane & Morrisey 2009)
		*Atrotorquata lineata*	Associated to salt-tolerant plants	(Kohlmeyer & Volkmann-Kohlmeyer 1993)
		*Candida deformans*	Insects, preserved fish, cured ham, yoghurt, spoiled food	(Knutsen et al. 2007)
		*Candida glaebosa*	Marine- and fresh-water, soil, wood, wine must, blue cheese, raw milk	mushroomobserver.org
		*Wallemia sebi*	Dust, food, mural paintings	Acc.No. AM159621
		*Peniophora cinerea*	Wood	(Chamuris 1991)
		*Euroticum amstelodami*	Soil, dust, indoor environments. Opportunistic pathogen	(Rakeman et al. 2005)
		*Exophiala pisciphila*	Saprophite found in soil, water, plants, decaying wood material	(Figel et al. 2013)
		*Trichosporon* spp*.*	Cheese	(Borelli et al. 2006; Vasdinyei & Deak 2003)
2200	14	*Kluyveromyces marxianus*	Raw milk, yogurt, cheese. Potential probiotic	(Hatoum et al. 2012; Jacques & Casaregola 2008; Lane & Morrisey 2009)
		*Debaryomyces hansenii*	Marine environment, brine, cheese, floor, walls, hands and equipments in cheese plants. Potential probiotic	(Hatoum et al. 2012; Jacques & Casaregola 2008; Welthagen & Viljoen 1998)
		*Candida deformans*	Insects, preserved fish, cured ham, yoghurt, spoiled food	(Knutsen et al. 2007)
		*Candida catenulata*	Cheese, floor and walls of cheese plants. Agent of fungemia	(Larsson & Őrstadius 2008; Jacques & Casaregola 2008; Radosavljevic et al. 1999; Welthagen & Viljoen 1998)
		*Candida pararugosa*	Widespread in the environment, milk and cheese. Opportunistic pathogen.	(Giammarco et al. 2004; Nakagawa et al. 2004), Acc No GQ458032
		*Candida incospicua*	Beer, milk and cheese, animal feces. Opportunistic pathogen	(Borelli et al. 2006)
		*Aspergillus versicolor*	Plants, cheese, marine water, starchy food. Opportunistic pathogen	(Torres-Rodríguez et al. 1998)
		*Pichia subpelliculosa*	Raw milk cheese, fermented olives	(Borelli et al. 2006), Acc No JQ419793
		*Galactomyces geotrichum*	Soil, insects, milk, cheese. Plant pathogen	cbs.knawl.nl
		*Torulaspora delbrueckii*	Raw milk, cheese, equipment in cheese plants. Potential spoilage yeast. Potential probiotic	(Borelli et al. 2006; Hatoum et al. 2012; Jacques & Casaregola 2008; Welthagen & Viljoen 1998)
		*Psathyrella lutensis*^*a*^	Soil, wood, animal feces	(Larsson & Őrstadius 2008)
		*Macrophomina phaseolina*	Soil. Plant pathogen	Acc No. GU046904
		*Funneliformis mosseae*	Mutualistic symbiont of plants.	Acc No. JN847444 tolweb.org
		*Yarrowia lipolytica*	Food, cheese, drinks. Potential probiotic	(Hatoum et al. 2012; Jacques & Casaregola 2008)
		*Clavispora lusitaniae*	Equipment and hands in cheese plants, fruit, cheese. Opportunistic pathogen.	(El-Sharoud et al. 2009; Jacques & Casaregola 2008; Welthagen & Viljoen 1998)
		*Pachyphloeus virescens*^*a*^	Soil, truffle	Acc No EU543198
		*Peronospora pulveracea*	Water. Plant pathogen	(Gőker et al. 2009)
		*Galactomyces* spp.	Soil, fruit, milk, cream, cheese	cbs.knawl.nl
		*Cryptococcus* spp.	Air, cheese	(Welthagen & Viljoen 1998)
		*Rhodotorula* spp.	See sediments, air, cheese	(Welthagen & Viljoen 1999)
<700m	9	*Kluyveromyces marxianus*	Raw milk, yogurt, cheese. Potential probiotic	(Hatoum et al. 2012; Jacques & Casaregola 2008; Lane & Morrisey 2009)
		*Debaryomyces hansenii*	Marine environment, brine, cheese, floor, walls, hands and equipments in cheese plants. Potential probiotic	(Hatoum et al. 2012; Jacques & Casaregola 2008; Welthagen & Viljoen 1998)
		*Candida pararugosa*	Widespread in the environment, milk and cheese. Opportunistic pathogen.	(Giammarco et al. 2004; Nakagawa et al. 2004), Acc No GQ458032
		*Candida parapsilopsis*	Raw milk cheese	(Borelli et al. 2006)
		*Galactomyces geotrichum*	Soil, insects, milk, cheese. Plant pathogen	cbs.knawl.nl
		*Exophiala dermatitidis*	Forest, water, wet indoor environments. Cutaneous infections. Neurotrophic	(Heinrich et al. 2013; Sudhadham et al. 2008)
		*Priceomyces carsonii*	Fermenting red wine	Acc No. JX456534

^a^ Macroscopic fungus.

A total of 31 fungal species have been retrieved, in addition to 5 genera not resolved at the species level, and a new sequence deposited in the GenBank under the Acc. No. KC954152. This sequence has been detected in a milk sampled at 1400 m and its best hit is the soil fungus JQ311712 (87% homology).

The 1400 m class yielded 10 species (Table 1) plus 1 unresolved genus and the new sequence KC954152, whereas at 1800 m 8 different species and 1 unresolved genus were observed. For milks sampled at 2200 m, 17 species and 3 genera as shown in Table 1 were obtained.

The most represented genus is *Candida*, with 6 different species, the majority of which (6 over 8 observations) retrieved at altitudes ≥ 1800 m. The genera *Exophiala*, *Aspergillus* and *Pichia* are respectively represented by 2 species (*E. pisciphila*, *E. dermatitidis, A. penicilloides*, *A. versicolor*, *P. membranifaciens*, *P. subpelliculosa*) without an altitude-specific segregation.

Species found at various altitudes comprehend *Kluyveromyces marxianus,* that resulted widespread as well as *Debaryomyces hansenii* (except for the 1800 m class). *Exophiala pisciphila* and *Atrotorquata lineata* characterized the intermediate classes (1400 and 1800 m) while *Candida deformans* was sampled at the highest altitudes (1800 and 2200 m).

In general, the identified species are mainly yeasts, followed by moulds and by 2 species of macroscopic fungi (signaled in Table 1).

The analysis of the D1/D2 domains of 9 raw milks sampled from herds at bottom of valleys (< 700 m) allowed to detect 7 species (Table 1). Four of them are shared with Alpine pastures: *K. marxianus, D. hansenii*, *Galactomyces geotrichum, C. pararugosa*.

### Fungal species identified by the various barcodes

Table 2 reports the list of the species detected by each of the 3 barcodes. A total of 21 species and 5 unresolved genera have been identified by the ITS1 barcode and 13 species by D1/D2, in addition to the new sequence KC954152. Finally, TUB discriminated 3 species.

**Table 2:** **Fungal species detected by the different barcodes**

Barcode	Identity	Number of milks	% similarity	Accession number
**ITS1**	*Kluyveromyces marxianus*	4	100	HE650691
	*Debaryomyces hansenii*	1	100	JQ912667
	*Galactomyces geotrichum*	2	100	AJ876893
	*Candida pararugosa*	2	98	GQ458032
	*Candida catenulata*	2	100	GU246267
	*Candida glaebosa*	1	98	JQ697543
	*Candida deformans*	3	99	AM279256
	*Candida incospicua*	1	99	AB179767
	*Atrotorquata lineata*	6	100	AF009807
	*Aspergillus versicolor*	1	99	JX526463
	*Aspergillus penicilloides*	1	100	DQ336711
	*Psathyrella lutensis*	1	97	DQ389685
	*Macrophomina phaseolina*	1	99	GU046904
	*Funneliformis mosseae*	1	100	JN847444
	*Yarrowia lipolytica*	1	100	AF335962
	*Clavispora lusitanae*	1	99	EF151449
	*Peronospora pulveracea*	1	100	FJ384778
	*Wallemia sebi*	1	100	AM159621
	*Peniophora cinerea*	1	100	GU062269
	*Pichia membranifaciens*	1	98	FM178293
	*Colleotothricum gloesporoides*	1	100	HQ645079
	*Galactomyces sp.*	1	100	DQ286062
	*Cryptococcus sp*	1	100	JQ327851
	*Rhodotorula sp*	1	99	DQ643075
	*Trichosporon sp*	1	100	JX270559
	*Phoma sp.*	1	100	JF912667
**D1/D2**	*Kluyveromyces marxianus* group 1	3 ^a^	100	HQ436414
	*Kluyveromyces marxianus* group 2	1+2 ^a^	100	HQ396523
	*Kluyveromyces marxianus* group 3	2 ^a^	100	FJ896141
	*Debaryomyces hansenii*	2 ^a^	100	HE681104
	*Galactomyces geotrichum*	1 ^a^	100	JX649971
	*Candida pararugosa*	1 ^a^	100	AB741061
	*Candida catenulata*	1 ^a^	100	FJ627977
	*Candida glaebosa*	1	99	EU327105
	*Candida parapsilopsis*	1 ^a^	100	AB741061
	*Pichia subpelluculosa*	1	100	JQ419793
	*Torulaspora delbrueckii*	1 ^a^	98	HE799671
	*Pachyphloeus virescens*	1	100	EU543198
	*Euroticum amstelodami*	1 ^a^	100	AY213699
	*Exophiala dermatitidis*	1 ^a^	100	JN391399
	*Priceomyces carsonii*	1 ^a^	95	JX456534
	Uncultured soil fungus^b^	1 ^a^	87	JQ311712
**TUB**	*Ophiocordyceps oxycephala*	1	100	EU604145
	*Alternaria solani*	1	100	JQ672057
	*Exophiala pisciphila*	2	97	JN112495

^a^Obtained by direct sequencing.

^b^Sequences deposited in the database.

Six species were identified by both ribosomal barcodes. In detail: *K. marxianus*, that resulted among the most abundant species, has been observed 4 times by ITS1 and 8 times by D1/D2 that also allowed to distinguish 3 sequence variants, characterized by in-del polymorphisms at the 26S rRNA locus as detailed in section 3.3. Other species identified by both barcodes comprehend, in order of abundance: *D. hansenii*, *G. geotrichum*, *C. pararugosa, C. catenulata, C. glaebosa*.

The ascomycetous *Atrotorquata lineata* is the most abundant species retrieved by a single barcode (ITS1), with a total of 6 observations.

### Three variants of *K. marxianus* sequences with in-del polymorphisms in domains D1/D2

Direct sequencing of 7 PCR products and of one cloned amplicon for the D1/D2 barcode revealed the presence of 3 variant sequences for *K. marxianus* carrying in-del polymorphisms in milk sampled at different altitudes. The first variant, 100% homologous to HQ436414 (Table 2), was retrieved in 2 milks sampled at 2200 m and in 1 at 1800 m. The second sequence (100% homology with HQ396523) characterized 1 sample at 2200 m and 1 at 1800 m, while the last one (100% homology with FJ896141) was found in 2 milks sampled at the bottom of valleys. The first group of sequences is characterized by the deletion of 2 nucleotides (G and A, respectively at positions 779 and 784 of HQ396523), while the third set has one deleted T as compared to groups 1 and 2 (see position 772 of HQ396523). These polymorphisms fall in the 5’ end of the region amplified by primers NL1/NL4 corresponding to the D1 domain of the 26S rRNA (De Rijk et al. 1997). Predictions performed either on the single sequences and on the consensus derived from the alignment showed that the retrieved in-del do not lead to significant changes in the stem-loop structure of the domain, that appears to have a conserved secondary structure in all the described variants (secondary structures not shown).

## Discussion

Mountainsides have often been the subject of biodiversity and biogeography studies, with several papers focused on changes of microbial diversity with elevation (Singh et al. 2012; Wang et al. 2012). On the other hand, altitude has been demonstrated to be among the pasture-specific features that more deeply influence cheese characteristics (Martin et al. 2005). The present paper combines these aspects by exploring the diversity of fungal consortia inhabiting cow raw milk sampled at different altitudes in Alpine valleys of Valle d’Aosta region and used for producing the Fontina PDO cheese. To this aim, we performed a multilocus sequencing at barcodes commonly in use for the identification of fungal species, integrated by an intermediate cloning step when necessary. This allowed to detect also rarer species thus enriching the spectrum of observations, and to resolve multiple band patterns.

In addition to fungi, the used barcodes reproducibly identified a well-defined group of bacterial, plant and metazoan sequences. Bacterial sequences were retrieved by all the barcodes in 16 different milk samples. The more represented bacteria resulted the environmental species *Escherichia coli*, *Enterobacter cloacae*, and *Macrococcus caseolyticus*, already isolated from cow’s milk and organs and from food-processing factories (Baba et al. 2009). Plants sequences were identified by ITS1 and TUB in 10 milk samples and mainly referred to grasses used to feed animals (*Lolium multiflorum*, *L. perenne*), flowering plants (e.g., *Achillea wilsoniana, Dactylis glomerata*), and wheat (*Triticum turgidum*). Finally, a sequence belonging to the cnidarian species *Chromonephthea franseni* was retrieved in 4 milks by ITS1. Alimentation and beverage are the plausible origins for all these sequences that have been retrieved in milks sampled at all altitudes. These results reflect known characteristics of most barcodes, especially the ribosomal ones. For example, ITS2, even if designed on fungal DNA, has proven to be effective for the identification of both plants and animals species and as such has been suggested as a possible “universal barcode” (Yao et al. 2010).

The fungal species detected in the analyzed raw milks are 31, in addition to 5 unresolved genera and a new sequence. ITS1 identified the majority of them, followed by D1/D2 and TUB. A short description of known sources and significance is provided in Table 1. Most species are yeasts, followed by moulds and by 2 macroscopic fungi (*Psathyrella lutensis* and the truffle *Pachyphloeus mosseae*). Among yeasts, it is worth mentioning two species of black or “melanized” yeasts belonging to the genus *Exophiala*. Black yeasts are stress-tolerant microorganisms, adapted to extreme habitats (Cantrell et al. 2011) and the subject of growing biotechnological and medical interest (Gunde-Cimerman et al. 2011). Some species have recently been reported in meat-processing facilities (Thanh et al. 2012) and in food as tropical fruit (Sudhadham et al. 2008) and cheese (Panelli et al. 2012). The genus *Exophiala*, composed by thermo- and acido-tolerant species, is a common inhabitant of many indoor and outdoor environments, especially of water, wet niches and forests. *Exophiala dermatitidis*, identified in a milk sampled at the bottom of valleys, has been recognized as the cause of infections in otherwise healthy patients in some tropical regions of South-Western Asia (Sudhadham et al. 2008).

The majority of the identified yeast species are common and known inhabitants of the dairy environment that play key roles in cheese-making processes thanks to their physiological and biochemical features (Jacques & Casaregola 2008). Among these, *K. marxianus* resulted the most abundant species in the Alpine raw milks and was retrieved at all the altitudes, even if the 3 sequence variants identified by D1/D2 seemed to have a more “clustered” distribution. This yeast is common in raw milk (Fleet 2007). It is also a predominant species in cheeses, both in those made from raw milk (Bai et al. 2010; El-Sharoud et al. 2009; Laurenčic et al. 2008) where it often dominates the yeast mycoflora, and in those produced using pasteurized milk (Giannino et al. 2011; Welthagen & Viljoen 1998; Welthagen & Viljoen 1999).

Other species that we found with minor frequency in the Alpine raw milk ecosystem comprehend *D. hansenii*, *Torulaspora delbrueckii, C. incospicua, C. pararugosa, C. glaebosa, G. geothricum, P. subpelliculosa,* all of which already reported in milk (Borelli et al. 2006; Jacques & Casaregola 2008). Another group is constituted by *C. catenulata, C. parapsilopsis, P. membranifaciens*, *A. versicolor*, and *Yarrowia lipolytica*, *Clavispora lusitanae, Rhodotorula* spp., *Trichosporon* spp., *Phoma* spp., *Cryptococcus* spp., already reported in various cheeses (Bai et al. 2010; Hocking & Faedo 1992; Jacques & Casaregola 2008; Montagna et al. 2004; Vasdinyei & Deak 2003; Viljoen & Greyling 1995; Welthagen & Viljoen 1999). *Candida* spp. was the most represented genus, with 6 different species the majority of which (6 over 8 observations) found at altitudes ≥ 1800 m.

Interestingly, one of the more abundant species, the environmental ascomycetous *A. lineata* (retrieved in 6 milks sampled at 1400 and 1800 m) has never been put in relation with food nor with the dairy ecosystem. It is a salt-tolerant food decomposer, inhabitant of salt-marsh areas of South-Eastern USA where it is found in association to the plant *Juncus roemerianus* (Kohlmeyer & Volkmann-Kohlmeyer 1993). To our knowledge, this is the first report of this species outside North-America. Its geographical distribution and ecology need to be updated as well as its possible roles in relation to human alimentation.

Finally, other species have never been reported in milk and are in general environmental fungi. Many of them result associated with plants: pathogens (e.g., *Colletothricum gloesporioides*, *Alternaria solani*, *Macrophomina phaseolina*, *Peronospora pulveracea*), mutualistic symbionts that form arbuscular mychorrhizae (*Funneliformis mossae*), or fungi retrieved in dust, soil, wood etc. (*A. penicilloides*, *Wallemia sebi*, *Peniophora cinerea*, *Euroticum amstelodami*, *Macrophomina phaseolina*).

In summary, the fungal community of raw milk sampled on Alpine highlands pastures from Valle d’Aosta appears composed by many species of diverse environmental origin and significance, several of which never reported in milk. The most abundant resulted the dairy yeast *K. marxianus* and the ascomycetous *A. lineata* along with the *Candida* genus. The highest sampled altitude (2200 m) resulted rich in fungal diversity (17 species vs. 8 at 1800 m, with similar numbers of sampled individual pastures). This observation reflects what previously reported (Singh et al. 2012). These authors observed a “peak” in bacterial diversity at around 2500 m when sampling soil at elevation intervals from 1000 m, and explained these results on the basis of the coincidence with the precipitation maximum that reduces UV and prevents drying of soil, thus increasing bacterial diversity.

## Conclusions

The work presented here enlarges the knowledge of fungal diversity in a primary alimentary matrix as raw milk. For the first time a microbial ecology study in the dairy field is performed with a focus on pasture altitude. On the other hand, for the first time, microbial ecology is included among factors, in the composition of Alpine pastures, that have the potential to shape the properties of milks and cheeses from animals grazing highland pastures, together with the already described physical, chemical and botanical variables (Buchin et al. 1999; Martin et al. 2005).
